# The embedding of stress: mitophagy as a mechanism for the central nervous system (CNS) programming and lifelong disease vulnerability

**DOI:** 10.3389/fcell.2026.1808059

**Published:** 2026-07-15

**Authors:** Meiling Wang, Ziyan Zhang, Zhou Sha, Fengfeng Zhang, Kaifan Bao

**Affiliations:** 1 Department of Immunology, School of Medicine, Nanjing University of Chinese Medicine, Nanjing, China; 2 Reproductive Medicine Center, The Central Hospital of Enshi Tujia and Miao Autonomous Prefecture, Enshi, China; 3 School of Life Science, Chongqing University, Chongqing, China

**Keywords:** CNS, epigenetic reprogramming, lifespan, mitophagy, psychological stress

## Abstract

Growing epidemiological evidence shows that psychological stress is strongly associated with pathogenesis in modern society a correlational finding). Its effects on CNS are highly age- and cell-type specific. This review outlines how stress regulates mitophagy and dynamically disrupt CNS homeostasis across different life stages, including fetal development, adolescence, and aging, as well as across distinct cell types such as neurons, astrocytes, and microglia. These alterations ultimately increase susceptibility to neurodevelopmental and neurodegenerative disorders.): We propose an integrated “cell–age–stress” framework (Hypothesis 1) that highlights the bidirectional crosstalk between stress-induced mitochondrial damage and nuclear epigenetic reprogramming. We also present a “lifespan intervention hypothesis” (Hypothesis 2) and discuss precision fine-tuning strategies targeted to specific life stages and cell types, offering new directions for neuroprotective therapies.

## 1 Introduction

Psychological stress has become a pervasive challenge in modern society, disrupting neurophysiological homeostasis through multiple signaling pathways (Madore et al., 2020; Haas et al., 2022). Neuroimaging studies have demonstrated that maternal and early-life stress induce structural and functional alterations in several brain regions, including the temporal lobe, frontal lobe, and amygdala (Bartlett et al., 2019; Van den Bergh et al., 2020). These adverse effects can occur throughout the lifespan. Prenatal maternal stress, social adversity, abuse, emotional neglect, malnutrition, and exposure to violence have all been associated with impaired neural development (Sanguino-Gómez and Krugers, 2024). In addition to its links to altered brain development, psychological stress has been correlated with accelerated cellular senescence (Carrier et al., 2021), and is associated with the aggravation of age-related neurodegenerative processes. Consequently, chronic stress has been implicated in the pathogenesis of various psychiatric and neurological disorders, including psychiatric disorders such as depression, Generalized Anxiety Disorder (GAD), Obsessive-Compulsive Disorder (OCD), Post-Traumatic Stress Disorder (PTSD), and neurodegenerative diseases such as Alzheimer’s Disease (AD) and Parkinson’s Disease (PD) (Maercker et al., 2022; Knezevic et al., 2023; Girotti et al., 2024).

Mitophagy is a specialized form of autophagy that selectively removes damaged or dysfunctional mitochondria. By doing so, it preserves organelle integrity and supports the generation of new mitochondria. Dysregulated mitophagy is implicated in numerous diseases. Mounting evidence shows that mitophagy is regulated in a highly cell-specific manner, especially in neurons. Neuronal mitophagy directly maintains circuit integrity and modulates vulnerability to neurodegeneration (Lou et al., 2020; Zhang et al., 2021). In the CNS, mitophagy is tightly controlled and dynamically balanced. Moderate mitophagy effectively clears stress-induced mitochondrial damage and sustains homeostasis in neurons and glia (Lou et al., 2020; Zhang et al., 2021; Picca et al., 2023). In contrast, insufficient mitophagy leads to the buildup of damaged mitochondria, elevated Reactive Oxygen Species (ROS) production, reduced Adenosine Triphosphate (ATP) synthesis, mitochondrial dysfunction, and eventual apoptosis (Su et al., 2023).

Stress initiates mitophagy via two main pathways: the ubiquitin-dependent PTEN-induced Kinase 1 (PINK1)-an E3 ubiquitin ligase (Parkin) pathway and the ubiquitin-independent pathway mediated by BCL2 interacting protein 3 (*BNIP3*), NIP3-like protein X (NIX), and FUN14 domain containing 1 (FUNDC1). Under stress, mitochondrial import through the Translocase of the Outer Mitochondrial membrane (TOM)/Translocase of the Inner Mitochondrial membrane (TIM) complex is impaired, causing PINK1 to accumulate and activate on the outer mitochondrial membrane (Heo et al., 2015; Artyukhova et al., 2019; Eldeeb et al., 2024). Parkin subsequently polyubiquitinates proteins on the outer mitochondrial membrane (Li et al., 2023). These ubiquitin chains are recognized by autophagy receptors, including Sequestosome one (p62/SQSTM1), Optineurin (OPTN), Nuclear Dot Protein 52 (NDP52), Tax1-binding protein 1 (TAX1BP1). Through their microtubule-associated protein 1 Light Chain 3 (LC3) interacting regions (LIR) (Panigrahi et al., 2020; Li J. et al., 2022; Nguyen et al., 2023), these receptors tether damaged mitochondria to the phagophore and facilitate autophagosome formation. The phagophore engulfs damaged mitochondria to form a double-membrane autophagosome, which fuses with lysosomes to form an autolysosome. Within the autolysosome, hydrolases degrade mitochondrial components, completing the removal of damaged organelles (Li A. et al., 2022). The BNIP3/NIX/FUNDC1 pathway functions in a ubiquitin-independent manner. These outer mitochondrial membrane proteins directly interact with LC3, thereby facilitating the recruitment of mitochondria to the phagophore (Lin et al., 2021; Wu et al., 2021).

Psychological stress is a major disruptor of mitophagic homeostasis. Stress signaling—particularly via Hypothalamic-Pituitary-Adrenal (HPA) axis activation—directly impairs mitochondrial function and epigenetically regulates the expression of mitophagy-related genes, altering pathway efficiency (Levy et al., 2022; Sanacora et al., 2022; Ashwani et al., 2025). In addition, altered methylation and hydroxymethylation of mitochondrial DNA (mtDNA) are closely linked to disease-associated mitochondrial dysfunction and may retrogradely modify nuclear epigenetic states (Stoccoro and Coppedè, 2021; Mostafavi Abdolmaleky and Zhou, 2024). Together, these observations support a unifying hypothesis: in the nervous system, mitophagy acts not only as a housekeeping process for organelle quality control but also as a dynamic regulator of stress adaptation.

In this review, we outline the life-cycle progression of stress-induced mitophagy. During the fetal period, maternal stress programs mitophagy through epigenetic modifications to drive compensatory adaptation (Manoli et al., 2007; Choi et al., 2021). In adolescence, the combination of heightened brain plasticity and ongoing stress can destabilize this balance. Mitochondria-to-nucleus metabolic-epigenetic crosstalk then becomes maladaptive, promoting mitochondrial dysfunction and increasing susceptibility to neuropsychiatric disorders (Kim et al., 2013; West et al., 2015; Duan et al., 2021; Zhou et al., 2024). In late life, cellular aging and cumulative stress drive systemic failure of mitophagy, sharply elevating the risk of neurodegenerative diseases (Redmann et al., 2016; Knuppertz et al., 2017; Chamoli et al., 2023). Therefore, understanding how stress reshapes mitophagy across the lifespan is critical for deciphering age-dependent vulnerability to neuropsychiatric and neurodegenerative conditions.

## 2 Psychological stress converges on mitochondria

### 2.1 Direct effects of excess glucocorticoids on mitochondrial oxidative stress and dynamics

Stress activates the HPA axis and triggers excess Glucocorticoid (GC) synthesis and release (O’Connor et al., 2021). In CNS, adverse prenatal environments also elevate GCs levels (Abellán-Álvaro et al., 2021; Liu Y. et al., 2024), reduce Glucocorticoid Receptor (GR) and Mineralocorticoid Receptor (MR) expression, and suppress Brain-Derived Neurotrophic Factor (BDNF) transcription, synapse formation, and BDNF-dependent dendritic growth (Chan et al., 2018). A study of 187 abused children aged 7–17 years revealed widespread HPA axis dysfunction characterized by increased daily cortisol output (Marques-Feixa et al., 2023). Consistently, chronic high stress and elevated cortisol in late childhood or adolescence positively correlate with an increased risk of psychosis (Cullen et al., 2022) (an epidemiological correlation). However, it is important to note that these clinical observations primarily reflect correlation rather than direct causation. In older adults, impaired neuroendocrine–immune crosstalk is associated with heightened vulnerability to emotional stress, and high cortisol levels are linked to cognitive decline and dementia In older adults, impaired neuroendocrine–immune crosstalk heightens vulnerability to emotional stress, and high cortisol levels are linked to cognitive decline and dementia (Muntsant and Giménez-Llort, 2022).

GCs regulate multiple energy-consuming processes, including mitochondrial energy metabolism. GCs enhance their own anti-inflammatory effects by promoting Tricarboxylic Acid (TCA) cycle–mediated mitochondrial metabolic reprogramming (Auger et al., 2024). The effects of GCs on mitochondria are biphasic: short-term exposure supports mitochondrial biogenesis and boosts respiratory chain enzyme activity, whereas prolonged exposure impairs respiratory chain function, reduces ATP production, increases ROS generation, and distorts mitochondrial structure (Kokkinopoulou and Moutsatsou, 2021). In osteoblast death, GCs trigger excessive ROS and disrupt mitochondrial homeostasis (Fan et al., 2025), suggesting a conserved mechanism in CNS cells. ROS selectively damages telomeres, inhibits telomerase, and activates the p53 pathway, which reduces mitochondrial biogenesis and represses peroxisome proliferator-activated receptor-γ coactivator-1α (PGC-1α) promoter activity (Baldelli et al., 2014; Lin and Epel, 2022; Figueiredo-Pereira et al., 2023). As a master regulator of mitochondrial biogenesis and oxidative metabolism, PGC-1α balances mitophagy and mitochondrial synthesis while limiting ROS accumulation. Notably, PGC-1α inhibition reduces NIX-dependent mitophagy, causes mitochondrial accumulation, and induces synaptic deficits in hippocampal neurons, SH-SY5Y cells, and ICR mice, marked by reduced synaptic density and impaired vesicle recycling (Choi et al., 2021). Therefore, we hypothesize that PGC-1α may be a critical molecular mediator through which GCs induce mitochondrial damage.

At the ionic level, studies have shown that ROS (e.g., hydrogen peroxide) regulate the trafficking of AMPA subtype of synaptic glutamate receptors (AMPARs) by modulating activity-dependent calcium signaling (Doser et al., 2020). When neurons are excited, Ca^2+^ rapidly enters mitochondria, followed by an increase in ROS production. This ROS signaling leads to the downregulation of excitatory synaptic channel expression, indicating that mitochondrial Ca^2+^ and ROS act synergistically to regulate synaptic excitability, positioning mitochondrial membrane potential (MMP) at the core of the regulatory network (Ahmed Selim and Wojtovich, 2025). MMP is crucial for the recruitment of Parkin in mitophagy. Low membrane potential leads to the selective recruitment of Parkin to mitochondria, thereby triggering further mitophagy. (Narendra et al., 2010). Based on this, we propose the hypothesis that ROS accumulation induced by GCs may affect MMP by increasing mitochondrial Ca2+ influx, thereby influencing mitophagy.

### 2.2 Stress-induced epigenetic modifications imprint mitochondrial function

Growing evidence indicates that maternal pre-conception stress is associated with excess GC release and mitochondrial oxidative stress, which correlates with intergenerational epigenetic reprogramming and persistent neurodevelopmental deficits in offspring (Maccari and Morley-Fletcher, 2007; Kingsbury et al., 2016). Building on these correlational findings, we speculatively hypothesize that such epigenetic marks—potentially mediated by dysregulated mitophagy—might participate in transgenerational feedback loops. However, whether these stress-induced epigenetic signatures are truly heritable across multiple generations without decaying remains a speculative hypothesis requiring rigorous transgenerational animal studies. In offspring of Holocaust survivors, maternal prenatal stress alters HPA axis sensitivity through DNA methylation of the GR gene (Chan et al., 2018). Parallel findings in rodent models show that poor maternal care induces hypermethylation of CpG islands in the GR promoter region (McEwen et al., 2016). Early-life trauma, including physical, sexual, or emotional abuse and neglect, is associated with sustained HPA axis abnormalities, including impaired GCs-mediated negative feedback and heightened cortisol reactivity in adulthood (Nikkheslat et al., 2020). Childhood maltreatment is strongly correlated with hypermethylation of exon 1F of the GR gene (NR3C1) in the hippocampus, reducing GR expression and amplifying stress-related depression- and anxiety-like responses (Cao et al., 2024). In stressed myocardium, activated NR3C1 disrupts calcium homeostasis and mitochondrial quality control by inhibiting glomulin (GLMN) (Cong et al., 2025), in clear cell renal cell carcinoma, NR3C1 induces mitophagy via the activating transcription factor 6 (ATF6)–PINK1/BNIP3 pathway (Yan et al., 2023). Although these findings have not yet been validated in CNS disorders, we propose the following hypothesis: NR3C1 methylation contributes to CNS disease pathogenesis through the downstream regulation of mitochondrial function and mitophagy. Further causal studies using cell-type-specific knockouts in neurons and glial cells are required to test this hypothesis.

The epigenetic regulator methyl-CpG-binding protein 2 (MECP2) mediates experience-dependent programming of HPA axis genes, including Corticotropin-Releasing Hormone (CRH) and Arginine Vasopressin (AVP). Hemizygous female MECP2 mice show anxiety-like behavior and reduced paraventricular nucleus activation, confirming MECP2’s role in HPA reprogramming (Abellán-Álvaro et al., 2021). This effect likely involves CpG island methylation driven by interactions between MECP2 and RNA polymerase II (Pol II) (Liu Y. et al., 2024). CRISPR-Cas9–edited MECP2 mutants show impaired mitophagy due to dysregulated BNIP3L expression (Zhou et al., 2026). In stroke models, lactylated MECP2 disrupts mitochondrial respiration, metabolic signaling, and neuroinflammation (Zhang et al., 2026). As a stress-responsive epigenetic regulator, MECP2 likely mediates stress effects by transcriptionally controlling downstream targets, including mitophagy-related genes.

In most neurodevelopmental syndromes, differentially methylated positions (DMPs) and regions (DMRs) are enriched in gene promoters and CpG islands, especially for genes involved in neurogenesis, synaptic signaling, and transmission (Levy et al., 2022). In AD, aberrant methylation of Amyloid Precursor Protein (APP), Presenilin 1 (PSEN1), Beta-site APP Cleaving Enzyme 1 (BACE1), Microtubule-Associated Protein Tau (MAPT), and Apolipoprotein E (APOE) coincides with histone deacetylation–dependent synaptic and memory impairment (De Plano et al., 2024). APOE is a major genetic risk factor for AD and regulates histone acetylation in neurons. APOE enriches histone H3 lysine 27 acetylation (H3K27ac) at immediate-early gene promoters to support memory consolidation (Li et al., 2021; Lee et al., 2023). APOE4 causes lysosomal lipid accumulation and blocked autophagic flux (Eran and Ronit, 2022; Lee et al., 2023), possibly through altered histone acetylation. In neurodegenerative diseases, mtDNA methylation and hydroxymethylation are altered, most prominently in the mitochondrial D-loop region, consistent with disease-related mitochondrial dysfunction (Stoccoro and Coppedè, 2021).

### 2.3 Stress leaves an initial molecular imprint on mitochondria via epigenetic modification

**FIGURE 1:**
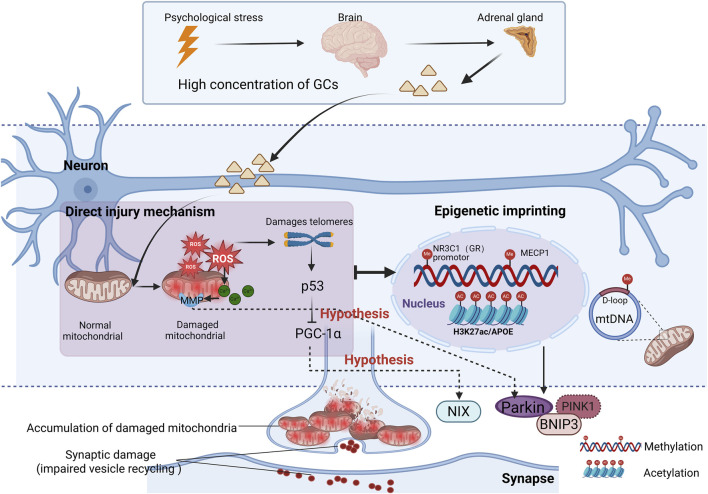
Psychological stress leaves an “initial molecular imprint” on mitochondria via oxidative stress and epigenetic reprogramming. Psychological stress overactivates HPA axis, leading to chronic excess release of GCs. In neurons, sustained high GCs directly impair the mitochondrial respiratory chain and trigger excessive ROS production. ROS accumulation is associated with telomere damage and activates the p53 pathway, which strongly inhibits PGC-1α, directly weakening NIX-dependent mitophagy. From the perspective of ion channels, we hypothesized that excessive accumulation of ROS leads to increased mitochondrial Ca2+ influx, which affects MMP expression and further disrupts Parkin recruitment. Along with acute oxidative injury, stress leaves profound and stable epigenetic imprints in both the nuclear and mitochondrial genomes. In the nucleus, stress induces hypermethylation of the GR gene (NR3C1) promoter and the epigenetic regulator (MECP2), and alters APOE-mediated histone modifications such as H3K27ac. These changes impair HPA axis negative feedback and disrupt downstream gene transcription. In mitochondria, aberrant methylation and hydroxymethylation occur in mtDNA, particularly in the D-loop region. Direct PGC-1α inhibition and long-term epigenetic changes converge to coordinately downregulate key mitophagy regulators, including PINK1, Parkin, NIX, BNIP3. This persistent molecular imprint irreversibly disrupts the mitochondrial quality control network, leading to massive accumulation of damaged mitochondria in synapses. Ultimately, this persistent molecular imprint is strongly correlated with neurodevelopmental and cognitive deficits, including reduced synaptic density and impaired vesicle recycling (epidemiological correlation). (Dashed lines indicate hypothesized pathways, whereas solid lines represent established mechanisms).

As illustrated in Figure 1, chronic psychological stress triggers excess GCs release, directly impairing mitochondrial function, increasing ROS accumulation, disrupting energy metabolism, and unbalancing mitochondrial dynamics, this process downregulates PGC-1α. At the ionic level, the accumulation of ROS leads to mitochondrial Ca^2+^ influx and changes in mitochondrial membrane potential (MMP), which may affect mitophagy (NIX, Parkin) and further impact the CNS. At a deeper level, stress leaves stable molecular imprints through epigenetic modifications in both the nuclear genome (e.g., NR3C1, MECP2 methylation, H3K27ac) and mitochondria (e.g., mtDNA methylation) (Stoccoro and Coppedè, 2021; Levy et al., 2022; Sanacora et al., 2022; Ashwani et al., 2025). These changes modulate expression of mitophagy regulators (e.g., PINK1, Parkin, NIX, BNIP3), establishing persistent mitochondrial alterations that increase cellular vulnerability to subsequent stress. The causal chain of this “initial molecular imprint” requires further testing in controlled experiments and longitudinal population studies. Together, these signaling and epigenetic events form the early mechanism by which stress “programs” mitochondrial function. Even after stress resolution, these mitochondrial and genetic imprints persistently alter cellular metabolism and autophagy capacity, laying the foundation for life-long dynamic changes in mitophagy.

## 3 Cellular heterogeneity of stress-induced mitophagy: distinct roles in neurons and glia

The CNS is highly vulnerable to pathological stress. Chronic stress drives profound biochemical alterations, synaptic remodeling, and neural dysfunction (Hyer et al., 2021). Within the complex brain microenvironment, neural network integrity depends on precise metabolic and immune crosstalk between neurons and glia (Soto et al., 2023). Mitophagy is regulated in a strongly cell-type-specific manner, yielding distinct outcomes across CNS cell populations.

### 3.1 Axonal/dendritic subdomain-specific mitophagy in neurons and its lifespan dynamics

Neuronal survival and complex electrophysiological activity rely heavily on ATP produced by mitochondria. Mitochondrial dysfunction is an early trigger of neuronal degeneration and death (Song et al., 2023; Zhao et al., 2024). The unique polarized structure of neurons restricts mitophagy to distinct subcellular regions. Most mitochondria in mature neurons localize to distal dendrites and axons, far from the lysosome-rich soma. The health and precise distribution of these local mitochondria directly determine synaptic integrity (Ge et al., 2020).

Under physiological conditions or mild acute stress, neurons maintain rapid mitochondrial turnover via local mitophagy (Duan et al., 2021). When focal mitochondrial damage occurs in hippocampal axons, mitochondrial Rho GTPase (Miro) is rapidly ubiquitinated and degraded. This blocks dynein-dependent retrograde transport and “traps” damaged mitochondria in place (Ashrafi et al., 2014; Ge et al., 2020). The outer membrane damage sensors PINK1 and Parkin then accumulate locally, together with LC3-positive autophagosomes and Lysosome-associated Membrane Protein 1 (LAMP1) - positive lysosomes, to clear damaged organelles *in situ* and suppress oxidative cascades (Martinez et al., 2017; Ge et al., 2020). A parallel PINK1–Parkin-independent pathway also operates. For example, mitochondrial E3 ubiquitin ligase 1 (MUL1/Mulan) can clear damaged mitochondria autonomously when PINK1 is absent (McWilliams et al., 2018).

Because neurons are post-mitotic and irreplaceable throughout life, mitophagy is essential for managing proteotoxic stress and maintaining homeostasis in distal compartments (Stavoe and Holzbaur, 2019), and it changes markedly with age. In mouse models of cerebral ischemia, ischemic stress or high exogenous GCs modify proliferation-associated 2G4 (PA2G4)/ErbB3-binding protein 1 (EBP1). PA2G4/EBP1 undergoes *in situ* ubiquitination, disrupting its interaction with p62 and inhibiting synaptic mitophagy, which restricts dendritic cytoskeletal development (Hwang et al., 2024). These findings suggest that GCs suppress axonal/dendritic mitophagy across multiple stages of neural development. Pregnancy is a critical window for neuronal maturation. High GCs levels caused by maternal stress may similarly inhibit dendritic and axonal mitophagy, impairing neuronal development and synapse formation, and thereby increasing vulnerability to later neurodevelopmental disorders.

### 3.2 Synaptic pruning by astrocytes and APOE4-Mediated autophagic flux blockade

Astrocytes are the most abundant glial cells in the CNS. They provide essential energetic support for synaptic transmission through lactate shuttling and glutamine secretion (Miyamoto et al., 2019).

Astrocyte proliferation, differentiation, and maturation peak from birth through late childhood in humans (corresponding to the first three postnatal weeks in rodents), a key period for brain development (Vivi et al., 2023). This window overlaps with active synaptogenesis. Astrocytes tightly regulate the balance between synapse formation and elimination, helping to establish functional neural networks (Vivi et al., 2023; Vivi and Di Benedetto, 2024). In mice, astrocytes target glutamatergic synapses and promote synaptic pruning via the Multiepidermal Growth Factor-like domain protein 10 (MEGF10) - mediated phagocytic pathway throughout development and adulthood. Knockdown of the *Drosophila* homolog of phosphatidylserine impairs synaptic pruning, supporting evolutionary conservation of this mechanism (Nelson and Broadie, 2025).

APOE4 is the strongest genetic risk factor for late-onset AD (De Plano et al., 2024). *In vitro* and pathological evidence link APOE4 to defective astrocytic mitophagy. In APOE4-carrying astrocytes, abnormal accumulation of cholesterol and unesterified lipids in lysosomes impairs lysosomal acidification (due to reduced V-ATPase activity). This sharply reduces the docking efficiency of autophagy adaptors LC3/p62 to damaged mitochondria and weakens lysosomal substrate degradation (Li et al., 2021; Lee et al., 2023). APOE4-driven lysosomal lipid overload and autophagic arrest disrupt mitochondrial homeostasis and promote reactive astrogliosis and neuroinflammation in the aging brain (Lee et al., 2023), reinforcing the central role of mitophagy failure in aging and AD pathogenesis.

### 3.3 Sentinel amplification of immune activation and “inflammaging” driven by impaired microglial mitophagy

Microglia are the resident immune cells of the CNS, acting as dual sentinels for environmental surveillance and synaptic pruning (Paolicelli et al., 2011). At the molecular level, collapse of microglial mitophagy triggers robust pro-inflammatory signaling. During chronic psychological stress or exposure to inflammatory toxins such as LPS, microglia show elevated mtDNA release and ROS production (Wang R. et al., 2024). Impaired mitophagy allows damaged mtDNA to leak into the cytosol, where it is detected by the pattern recognition receptor--cyclic GMP-AMP synthase (cGAS). This activates the downstream stimulator of interferon genes (STING)–interferon regulatory factor 3 (IRF3) pathway (West et al., 2015; Zhou et al., 2024). Concurrently, cytosolic ROS accumulation promotes assembly of the nucleotide-binding oligomerization domain-like receptor protein 3 (NLRP3) inflammasome, leading to robust maturation and release of pro-inflammatory cytokines including IL-1β and IL-18, and triggering a neuroinflammatory cascade (Thangaraj et al., 2020; Han X. et al., 2021; Wang H. et al., 2024; Zhou et al., 2024).

Immune dysregulation caused by mitophagy failure in microglia leaves distinct, age-specific pathological signatures across the lifespan. In the fetal period, external insults such as zinc oxide nanoparticles activate the E3 ubiquitin ligase mouse double minute 2 (MDM2), which ubiquitinates the mitochondrial contact site and cristae organizing system complex (MICOS)--Mic60 protein (Zhang et al., 2023). Prenatal exposure to stimuli such as caffeine causes intrauterine growth restriction in rats, leading to excessive microglial activation and synaptic pruning, ASD-like behaviors, and hippocampal damage (Wang T. et al., 2023). Single-nucleus RNA sequencing of whole brain or prefrontal cortex tissue reveals that microglia in aged brains upregulate inflammatory genes and downregulate homeostatic markers (Jin et al., 2025; Muralidharan et al., 2025), supporting a shift toward a pro-inflammatory phenotype with age. Quinolinic acid, a metabolite secreted by stressed microglia, is elevated in brain aging and age-related neurodegeneration. It impairs mitophagy and blocks mitolysosome formation (Dongol et al., 2023), locking microglia in a chronic pro-inflammatory state termed inflammaging. In this state, sustained cytokine release acts on nearby neurons and astrocytes, accelerating the deposition and spread of amyloid β (Aβ) and hyperphosphorylated tau. This drives severe progression of neurodegenerative diseases including AD and PD in older individuals (Fang et al., 2019; Zhou et al., 2024).

**FIGURE 2:**
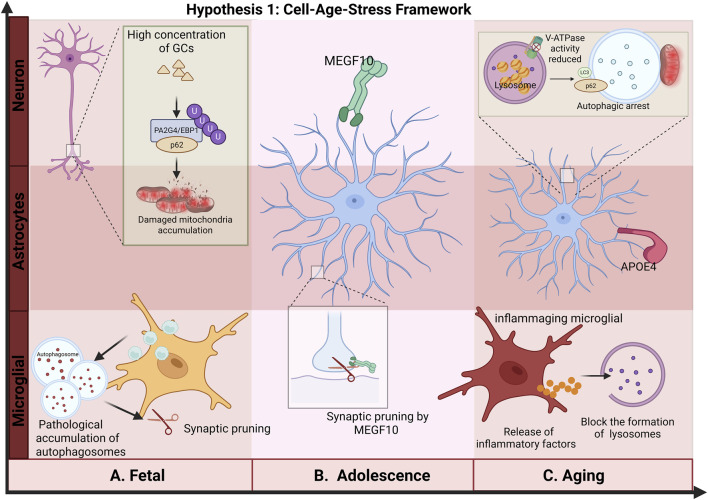
Regarding the three-dimensional regulatory framework of “cell-age-stress”. Hypothesis 1: The “cell-age-stress” three-dimensional regulatory framework. This integrated framework hypothesizes a three-dimensional cross-interaction between environmental stress load (Stress), life stage (Age), and CNS cell types (Cell type). In the three-dimensional coordinate network, the pathological effects of stress exhibit extremely strong spatiotemporal specificity. **(A)** Fetal: High GCs and other stress during pregnancy alter the *in situ* ubiquitination of PA2G4/EBP1 in neurons, blocking its binding to p62, and inhibiting synaptic mitophagy. Simultaneously, abnormal fetal environmental stimuli activate the MDM2 pathway within microglia, leading to pathological accumulation of autophagosomes, abnormal activation of glial cells, and exaggerated synaptic pruning. **(B)** Adolescence: In the face of chronic stress, the phagocytic function mediated by MEGF10 on the surface of astrocytes is abnormally upregulated, inducing excessive synaptic pruning and microenvironmental neuroinflammation. **(C)** Aging: Under the background of long-term stress accumulation and aging, microglia completely transform into a strong pro-inflammatory phenotype due to metabolic-dependent lysosome formation disorders. Concurrently, in astrocytes carrying the genetic risk gene APOE4, chronic inflammatory stress drives excessive lipid overload in lysosomes, leading to a decline in V-ATPase activity, which correlates with a sharp drop in the efficiency of docking and degradation of damaged mitochondria. The mitochondrial collapse of astrocytes and the inflammatory senescence of microglia mutually reinforce each other.

Collectively, we propose an integrated “cell–age–stress” three-dimensional regulatory framework (Figure 2). High prenatal GC stress inhibits synaptic mitophagy in neurons by altering *in situ* ubiquitination of PA2G4/EBP1 and disrupting p62 binding, potentially contributing to neurodevelopmental disorders such as ASD. Prenatal insults also activate MDM2, triggering excessive autophagosome accumulation, aberrant glial activation, and exaggerated synaptic pruning, which lead to ASD-like phenotypes and hippocampal injury. The pro-inflammatory microglial phenotype dominates in late life, driven by metabolite-dependent impairment of lysosome formation. In astrocytes carrying the APOE4 allele, chronic inflammatory stress promotes mitochondrial collapse, lysosomal lipid overload, reduced V-ATPase activity, and inefficient docking and degradation of damaged mitochondria, accelerating AD and PD pathogenesis. In adolescence, astrocytic MEGF10-mediated phagocytosis promotes neuroinflammation and excessive synaptic pruning, increasing vulnerability to affective disorders.

## 4 Dynamic changes in mitophagy and stage-specific neuropathology

### 4.1 Fetal epigenetic programming and ASD vulnerability

Maternal stress elevates GCs levels. GCs cross the placental barrier, enter fetal circulation, and activate GR. Maternal stress exerts dual effects on fetal brain development, depending on stress type and intensity.

Prolonged exposure to high glucocorticoid levels destabilizes PINK1 on the outer mitochondrial membrane through GR signaling, suppresses PGC-1α activity, and ultimately impairs mitophagy. Reduced PGC-1α impairs mitochondrial synthesis and selectively lowers mitophagy, culminating in synaptic and neuronal damage (Manoli et al., 2007; Kingsbury et al., 2016; Choi et al., 2021). On the other hand, prenatal stressors such as nicotine activate the general control nonderepressible 2 (GCN2) pathway in the placenta. The GCN2/ATF4 pathway senses mitochondrial stress and rewires neuronal metabolism by upregulating mitochondrial dysfunction markers and the epigenetic metabolite 2-hydroxyglutarate (2-HG) (Zhu et al., 2021). 2-HG is a mitochondrial metabolite best studied in cancer, its elevation reflects mitochondrial dysfunction or stress-induced metabolic remodeling. Lowering 2-HG alleviates stress-related neuronal deficits, while excess 2-HG worsens them (Hunt et al., 2019). In mouse models, maternal stress increases brain 2-HG, indicating concurrent collapse of mitochondrial and epigenetic homeostasis (Alhassen et al., 2021). Upon stress-induced GCN2/ATF4 activation, 2-HG triggers compensatory BNIP3/NIX-dependent mitophagy. Although this response attempts to restore mitochondrial balance, it also raises ROS and may interfere with normal fetal development, warranting further investigation (Zhu et al., 2021). In summary, fetal exposure to maternal stress disrupts mitochondrial homeostasis via two main routes: prolonged GC exposure strongly inhibits mitophagy, and it also affects the development of synapses. Whereas the GCN2/ATF4 axis drives complex compensatory mitophagy, whether this pathway is associated with the CNS needs further verification.

The intergenerational transmission of stress mainly increases the risk of offspring developing ASD (Chan et al., 2018). Key ASD-associated genes are functionally linked to mitophagy, including encoding gene of Parkin (*PARK2)*, the activating molecule in BECLIN-1-regulated autophagy (*AMBRA1*), WD repeat and FYVE domain-containing 3 (*WDFY3*) and Cln three requiring 9 (*CTR9*). *PARK2* is critical for synaptic function. *AMBRA1* encodes a mitophagy receptor containing an LC3-interacting region (LIR), heterozygous *AMBRA1* mutation in mice produces ASD-like behaviors (Kovacheva et al., 2025). *WDFY3* encodes an adaptor that promotes autophagosome–lysosome fusion, its mutation disrupts neural development and synaptic plasticity (Napoli et al., 2018; Napoli et al., 2021). In a study of 13 mostly neonatal patients with heterozygous *CTR9* variants, four were diagnosed with ASD (Meuwissen et al., 2022), consistent with *CTR9*’s conserved role in *Drosophila* neural development (Bahrampour and Thor, 2016). Meuwissen et al. also demonstrated that CTR9 regulates mitophagy and its association with neurodevelopment in yeast, consistent with this, *CTR9* contributes to mitophagy induction (Zheng et al., 2020) We therefore hypothesize that *CTR9* variants contribute to ASD pathogenesis by disrupting mitophagy. At present, although the association of the CTR9 gene with neurodevelopmental disorders is well-established (Meuwissen et al., 2022), the causal link between ASD-related genes and mitophagy remains to be validated.

### 4.2 Adolescent dynamic disruption and depression

Adolescence is a critical period for brain maturation. As cellular powerhouses, mitochondria shape synaptic formation and development across the CNS (Zhang Z. et al., 2024). Chronic social and environmental stress during adolescence activates transcription factor C/EBPβ in medial prefrontal cortical neurons, upregulates the mitochondrial fission protein dynamin-related protein 1 (DRP1), and triggers mitochondrial dysfunction, thereby increasing stress vulnerability (Haynes et al., 2024; Chen et al., 2025). Physiological DRP1 activation promotes mitochondrial fission and supports PINK1/Parkin-mediated mitophagy, this effect is amplified by PINK1-dependent DRP1 phosphorylation at Ser616 (Han et al., 2020). DRP1 function is bidirectional, and its dysregulation is pathogenic. On the one hand, DRP1 deletion or downregulation causes excessive mitochondrial fusion, ubiquitin and mitophagy marker accumulation, and neurodegeneration (Wang et al., 2020; Yi et al., 2022; Wen et al., 2025). On the other hand, under chronic stress, pathological DRP1 hyperactivation (high Ser616 phosphorylation, low Ser637 phosphorylation (Wang et al., 2020)) triggers excessive mitochondrial fragmentation, ATP depletion, and ROS overload (Lu et al., 2025). Excessive fission promotes LC3II and SQSTM1/p62 buildup, blocks Parkin recruitment to damaged mitochondria, inhibits autophagosome–lysosome fusion, and stalls mitophagy (Yi et al., 2022; Wen et al., 2025). Tight DRP1 control is therefore essential for mitochondrial homeostasis, and its dysbalance is a key driver of adolescent stress-induced neural injury. Mitochondrial dysfunction and impaired mitophagy disrupt neuronal function and synaptic transmission (Zhang Z. et al., 2024), particularly along the pathway from the basolateral amygdala (BLA) to the bed nucleus of the stria terminalis (BNST) (Kim et al., 2013; Duan et al., 2021), precipitating depression-like behaviors (Gardner and Boles, 2011; Gong et al., 2011; Liu Q. et al., 2024).

Adolescent stress also impairs glial function. Mitochondrial damage in astrocytes contributes to neuronal toxicity (Yang et al., 2022; Yang et al., 2023). Stress inactivates mitofusin 2 (*Mfn2*), inhibiting PI3K/Akt/mTOR-dependent mitophagy in astrocytes (Yang et al., 2023). These glial defects amplify neuronal injury and inflammation, collectively establishing depressive circuits.

### 4.3 Senescent mitophagy-lysosome axis collapse and AD/PD

Mitochondrial dysfunction is a hallmark of aging (Chamoli et al., 2023), and lysosome-dependent autophagic clearance supports longevity (Redmann et al., 2016; Knuppertz et al., 2017). Aging directly impairs lysosome biogenesis and function via the mechanistic target of rapamycin (mTOR)/transcription factor EB (TFEB) pathway (Zhong et al., 2022), reducing mitophagy and allowing damaged mitochondria to accumulate. Aging also disrupts the HPA axis, triggering chronic low-grade inflammation and elevated GCs. High GC upregulates serum/glucocorticoid-regulated kinase 1 (SGK1). Inhibiting glial SGK1 suppresses the DNA sensor cGAS–STING pathway, reverses proinflammatory glial phenotypes, and reduces cellular senescence and mitochondrial damage (Kwon et al., 2021). Reverse inference suggests that the increase in glutamate levels may activate the cGAS-STING pathway, thereby triggering an inflammatory response in glial cells. GCs worsen mitochondrial injury and promote cytosolic release of mtDNA (Wang R. et al., 2024), and activate the cGAS–STING–IRF3 pathway, triggering sustained type I interferon responses. The cGAS–STING pathway also retrogradely promotes TFEB nuclear translocation to enhance lysosome biogenesis (Xu Y. et al., 2025). Together, these observations suggest that aging-related GC elevation creates a self-reinforcing cycle involving cGAS–STING–IRF3 and TFEB, generating an “autophagy–inflammation vicious cycle”.

In the adult brain, quiescent neural stem cells contain abundant lysosomes and can generate neural progenitors. With aging, these cells develop lysosomal dysfunction, increasing protein aggregation, suppressing stem cell activation (Leeman et al., 2018), and causing mitochondrial buildup. PD involves degeneration of dopaminergic neurons, driven by Lewy bodies that inhibit complex I, increase ROS, block mitochondrial transport to lysosomes (Kinnart et al., 2024), and trigger mitochondrial dysfunction (Swerdlow and Wilkins, 2020; Klemmensen et al., 2024). Chronic stress and GC dysregulation increase PD risk and accelerate progression by amplifying mitochondrial injury and inflammation (Wang R. et al., 2024). PD-linked mutations in α-synuclein (*SNCA*) and leucine-rich repeat kinase 2 (*LRRK2*) disrupt lysosomal pathways (Borsche et al., 2021). *LRRK2* mutations increase mitochondrial fragmentation, reduce membrane potential, elevate calcium uptake, and block mitochondrial clearance in dendrites (Lizama and Chu, 2021). In aged *LRRK2*-R1441G mice, abnormal DRP1 signaling causes ubiquitinated mitochondrial buildup, lowers stress tolerance, and accelerates neuronal apoptosis (Liu et al., 2021). Heightened stress sensitivity, combined with aging-related HPA dysregulation and chronic GC exposure, promotes neurodegeneration. Defective PINK1/Parkin/DJ-1-mediated mitophagy underlies autosomal recessive PD. DJ-1 acts with PINK1/Parkin to recruit autophagy receptors such as optineurin, loss of this interaction blocks mitophagy (Imberechts et al., 2022).

AD is defined by extensive Aβ plaques and neurofibrillary tangles of hyperphosphorylated tau (Xie et al., 2022). Chronic stress and sustained high GC increase AD risk and accelerate cognitive decline by promoting Aβ deposition, tau hyperphosphorylation, and neuroinflammation (Zhuravleva et al., 2021; Burke et al., 2024). Impaired mitophagy initiation (e.g., low phospho-TBK1, phospho-ULK1) disrupts energy metabolism, exacerbates Aβ and tau accumulation, and triggers synaptic failure and cognitive impairment (Kerr et al., 2017; Fang et al., 2019; Han S. et al., 2021). Age-related lysosomal dysfunction is the primary barrier to mitophagy initiation (Tan and Finkel, 2023), consistent with this finding, restoring lysosomal function in AD models removes abnormal mitochondria and preserves synaptic structure (Han S. et al., 2021). Damaged mitochondria also impair microglial clearance of neurotoxic proteins and dysregulate cytokine release (Fang et al., 2019). This aligns with cGAS–STING-driven neuroinflammation induced by mitochondrial injury, forming a self-reinforcing pathological cycle linking mitochondrial–lysosomal dysfunction and neuroinflammation in age-related neurodegeneration.

**FIGURE 3:**
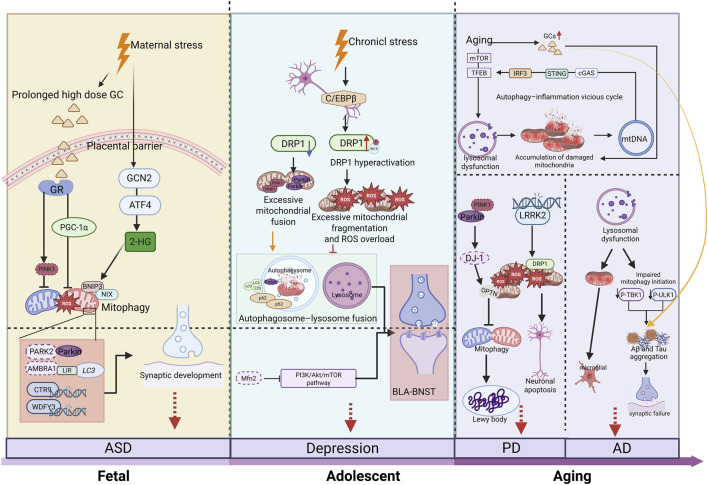
Dynamic changes in mitophagy across different life stages and neuropathological mechanisms in specific stages. Disruption of mitophagy homeostasis is strongly associated with the progression of distinct neuropsychiatric disorders throughout the lifespan, from fetal to elderly. Fetal epigenetic programming and susceptibility to (ASD): Elevated GCs levels caused by maternal stress can cross the placental barrier, activate GR receptors, reduce the stability of PINK1 and the expression of PGC-1α, thereby inhibiting mitochondrial biosynthesis and mitophagy functions, which correlates with impaired synaptic development. At the same time, stress factors can reprogram metabolism through the GCN2/ATF4 pathway in the placenta, increase 2-HG, trigger BNIP3/NIX-dependent mitophagy, and produce excessive ROS. In relation to this, mutations or downregulation of mitophagy related genes (such as PARK2, AMBRA1, WDFY3, CTR9) at the genetic level further increase the risk of synaptic development disorders and ASD. Dynamic homeostatic disruption during adolescent and depression: Chronic stress during adolescent activates C/EBPβ in neurons, leading to pathological overactivation of DRP1 (high phosphorylation at Ser616). This induces excessive mitochondrial fission and ROS overload, resulting in accumulation of LC3 and p62, blocking Parkin recruitment and autophagosome-lysosome fusion, thus stalling mitophagy. Conversely, DRP1 deficiency or downregulation causes excessive mitochondrial fusion and accumulation of mitophagy markers. Meanwhile, Mfn2 inactivation inhibits PI3K/Akt/ mTOR-dependent autophagy, causing neuronal damage and glial cell inflammation, collectively disrupting synaptic transmission and correlating with depressive-like behaviors (a correlational observation in animal models). Mitophagy-lysosomal axis collapse in aging and AD/PD: Aging and long-term exposure to high GCs directly impair lysosomes via the mTOR/TFEB pathway. Damaged mitochondria accumulate and release mtDNA into the cytoplasm, activating the cGAS-STING-IRF3 pathway, inducing persistent neuroinflammation, and forming an “autophagy-inflammation vicious cycle” with TFEB. In PD, mutations in genes such as LRRK2 and PINK1/Parkin/DJ-1 impede clearance of damaged mitochondria and promote Lewy body formation. In AD, high GCs promote Aβ deposition and Tau protein accumulation. Age-related lysosomal dysfunction leads to accumulation of mitochondrial damage and impaired microglial clearance. Additionally, weakened initiation signals of mitophagy (e.g., p-TBK1, p-ULK1) exacerbate Aβ deposition and hyperphosphorylation of Tau, ultimately resulting in severe synaptic failure. (Dashed arrows leading to disease states denote hypothesized causal links based on current correlational evidence).

As shown in Figure 3, the increase in GCs caused by maternal stress interferes with mitochondrial quality control through multiple mechanisms. Excessive GCs signals inhibit the PINK1/PGC1α pathway and damage mitophagy, resulting in impaired synaptic development. At the same time, the activated GCN2/ATF4 pathway induces compensatory mitophagy dependent on BNIP3/NIX, which has an unclear effect on synapses. However, related to this mechanism, mutations in mitophagy-related genes increase synaptic damage and the risk of ASD. During adolescence, chronic stress disrupts mitochondrial dynamics in a cell-specific manner. In medial prefrontal cortex neurons, stress-induced DRP1 hyperactivation promotes excessive mitochondrial fission and impairs autophagic flux by disrupting autophagosome–lysosome fusion. In parallel, suppression of Mfn2 signaling in astrocytes compromises mitochondrial network integrity and metabolic support. These alterations collectively impair synaptic remodeling and contribute to the development of depression-like phenotypes.

During aging, sustained high GCs cause lysosomal dysfunction, leading to collapse of the mitophagy–lysosome axis. Damaged mitochondria release mtDNA, which activates the cGAS–STING pathway and triggers persistent neuroinflammation, forming a self-reinforcing vicious cycle. This cycle ultimately promotes the accumulation of Aβ and hyperphosphorylated Tau in AD, as well as Lewy bodies in PD.

It is worth noting that the role of calcium in the pathogenesis of AD, ALS, and other diseases is beginning to emerge. Oligomeric Aβ exposure may be associated with mitochondrial calcium overload, and tau-mediated pathology is also linked to improper calcium handling, with mitochondrial calcium homeostasis disruption rendering neurons susceptible to excitotoxic damage (Verma et al., 2022). The previous description of Ca2+ influencing mitophagy through the regulation of MMP may explain this. Consistent with our hypothesis, primary cortical neurons transfected with LRRK2 exhibit increased calcium influx, which is mediated by the transcriptional upregulation of the mitochondrial calcium uniporter (MCU) and the mitochondrial calcium uptake one protein (MICU1) (Verma et al., 2017).

## 5 Mitochondria-to-nucleus mitokine feedback: epigenetic retrograde signaling

We have described how psychological stress elevates GCs release, disrupts mitochondrial homeostasis, and triggers a forward stress injury cascade marked by mitophagy failure and damaged mitochondria accumulation. This section focuses on the long-underappreciated reverse relationship: mitochondria-to-nucleus retrograde communication. When mitophagy is impaired across life stages, dysfunctional mitochondria act as active signaling hubs that retrogradely control nuclear gene expression (Li et al., 2025). Uncleared mtDNA, specific metabolites such as 2-HG, and local ROS translocate to or signal within the nucleus, dynamically reshaping the host epigenetic landscape.

### 5.1 Metabolites as epigenetic signals

Mitochondria generate multiple central cellular metabolites, including succinate and α-ketoglutarate (α-KG) from TCA cycle. These metabolites modulate DNA methylation by inhibiting α-KG-dependent DNA demethylases (Kinnaird et al., 2016).

2-HG is a mitochondrial metabolite that acts as a competitive inhibitor of α-KG and DNA demethylases (Chang et al., 2019). In anti-tumor T-cell immunity, 2-HG strongly increases the key histone methylation mark histone H3 lysine 27 trimethylation (H3K27me3) (Yang et al., 2025). Mechanistically, 2-HG competitively binds α-KG to inhibit the Jumonji-C (JmjC) family of lysine demethylases (KDMs), the Ten-Eleven Translocation (TET) family of 5′-methylcytosine hydroxylases, the Alkylation B (AlkB) family of Fe(II)/α-ketoglutarate-dependent dioxygenases, and the egg-laying defective nine (EGLN, also known as PHD) enzymes, leading to global histone hypermethylation (Hao et al., 2024).

These observations suggest that prenatal psychological stress-induced 2-HG elevation may drive hypermethylation of neurodevelopmental genes, thereby promoting ASD pathogenesis. In secondary glioblastoma, isocitrate dehydrogenase (IDH) mutations produce 2-HG, establishing a unique genome-wide hypermethylation profile termed the glioma CpG island methylator phenotype (G-CIMP), which drives tumorigenesis (Yamashita et al., 2019). In neural stem cells, 2-HG also reduces acetyl-CoA (Ac-CoA) levels (Furth et al., 2025). Ac-CoA is an essential substrate for histone acetylation--e.g., histone H3 lysine 27 acetylation (H3K27ac), mitochondrial changes in Ac-CoA can therefore retrogradely alter nuclear histone acetylation (Sun et al., 2024), further supporting our hypothesis.

### 5.2 Signaling roles of ROS

Dysfunctional mitochondria downregulate nuclear-encoded oxidative phosphorylation genes and activate the ROS–JNK mitochondria-to-nucleus retrograde pathway. This will trigger the formation of cytoplasmic chromatin fragments (CCFs) and induce senescence-related secretory phenotypes. CCFs are rich in heterochromatin markers, such as the methylation markers H3K9me3 and H3K27me3 (Vizioli et al., 2020). These findings suggest that the ROS-JNK pathway may be related to methylation changes.

While excessive ROS causes cellular damage, ROS also functions as a critical signaling molecule that activates multiple pathways, including those regulating epigenetic enzymes (Mostafavi Abdolmaleky and Zhou, 2024). High ROS levels modulate the activities of histone acetyltransferases (HATs), histone deacetylases (HDACs), and DNA methyltransferases (DNMTs), thereby epigenetically controlling gene expression (Li et al., 2024).

### 5.3 Signaling roles of mtDNA and mitochondrial RNA (mtRNA) fragments

Upon mitochondrial damage or dysfunction, mtDNA are released into the cytoplasm and may enter the nucleus (Wang et al., 2026). Mitochondria-derived long non-coding RNAs (mtlncRNAs) translocate from mitochondria to the nucleus, with RNA-binding proteins HuR and PNPT1 serving key roles in this process (Li et al., 2025). These mitochondrial nucleic acids are detected by pattern recognition receptors (PRRs) such as the cGAS–STING pathway, triggering inflammatory and interferon responses that indirectly reshape the epigenetic landscape (Ding et al., 2025; Gong et al., 2026; Wang et al., 2026).

Methylation and hydroxymethylation marks are widely present in the mtDNA genome (Zhang F. et al., 2024) and contribute to mitochondrial injury in multiple human diseases, including neurodegenerative disorders (Stoccoro and Coppedè, 2021). MtDNA N6-methyldeoxyadenosine (6 mA) modification plays critical roles in aging and neurodegeneration (Zhang L. et al., 2024; Zhang L. et al., 2025). Notably, in vascular cognitive impairment, the 6-mA methylation mediated by methyltransferase (METTL4) in mtDNA drives neuroinflammation through cGAS-STING activation (Gong et al., 2026).

**FIGURE 4:**
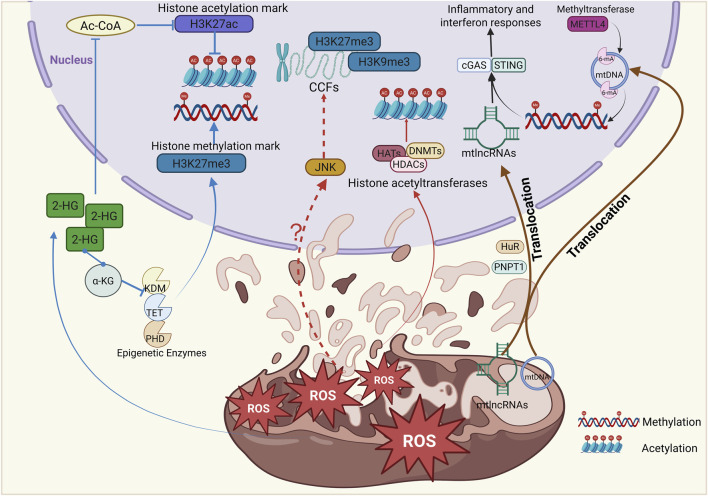
The epigenetic reverse signaling network between mitochondria and the nucleus (Mitokine feedback mechanism). In the case of impaired mitophagy, dysfunctional mitochondria act as an active signaling hub, releasing various mitochondrial-derived factors (Mitokines) to reverse-regulate gene expression and epigenetic status within the cell nucleus. The metabolite 2-HG produced by damaged mitochondria competitively binds to α-KG, strongly inhibiting demethylases (KDM, TET, PHD), promoting the accumulation of H3K27me3, resulting in global hypermethylation of the genome. The other side, 2-HG inhibits Ac-coA levels, acting by inhibiting H3K27ac on histone acetylation. Dysfunctioning mitochondria produce excessive ROS. ROS activates the cytoplasmic JNK pathway into the nucleus, inducing CCFs, which are rich in H3K27me3 and H3K9me3, leading to the hypothesis (indicated by dashed lines) that the ROS-JNK pathway has a role in promoting nuclear methylation. Additionally, ROS acts as a key signaling molecule, directly or indirectly regulating the activities of HATs, HDACs, and DNMTs, dynamically reshaping the epigenetic landscape. Mitochondrial damage leads to the leakage of mtDNA and mtlncRNAs into the cytoplasm. mtDNA is translocated to the nucleus, where methyltransferase METTL4 catalyzes 6-mA methylation of mtDNA, activating the cGAS-STING pathway and triggering neuroinflammation. At the same time, mtlncRNAs are directly translocated into the nucleus under the mediation of RNA-binding proteins HuR and PNPT1, also initiating the cGAS-STING signaling pathway, which is linked to inflammation and interferon response. (Solid lines represent experimentally validated molecular translocations, while dashed lines with question marks indicate speculative feedback mechanisms).

As shown in Figure 4, when mitophagy is impaired, dysfunctional mitochondria are no longer passive victims of cellular damage but become active signaling hubs that reshape the host’s epigenetic landscape through mitochondria-to-nucleus retrograde signaling, this feedback relies on three types of mitochondrial signals. Among these, stress-induced 2-HG plays two roles. On one hand, it competitively binds to α-KG and inhibits demethylases, promoting global hypermethylation. On the other hand, it lowers Ac-CoA levels to exert an inhibitory effect on histone acetylation.

Stress-induced mitochondrial damage activates the ROS-JNK retrograde signaling pathway from mitochondria to the nucleus, leading to the formation of CCFs and the development of senescence-associated secretory phenotype, and further epigenetic changes such as methylation. High ROS levels regulate the activity of HATs, HDACs, DNMTs, thereby controlling gene expression at the epigenetic level. Upon mitochondrial damage and dysfunction, mtDNA translocates into the nucleus, where methyltransferase METTL4 catalyzes 6-mA methylation of mtDNA to activate the cGAS-STING pathway and trigger neuroinflammation. The mtlncRNAs are shuttled into the nucleus with the assistance of RNA-binding proteins HuR and PNPT1, and also initiate inflammatory and interferon responses via the same cGAS-STING signaling cascade.

## 6 Therapeutic perspectives: precision interventions across the lifespan

Strategies targeting mitochondrial quality control across the lifespan must not blindly and globally activate mitophagy. Non-specific over-activation can cause severe adverse effects, such as damaging healthy mitochondria or triggering cell death. Therefore, based on the “cell-age-stress” three-dimensional framework discussed earlier, we propose a “lifespan intervention hypothesis.” This involves targeted interventions on the “stress-mitophagy-CNS regulatory axis” during critical developmental windows to develop individualized and spatiotemporally specific strategies.

### 6.1 The fetal stage and ASD: blocking endocrine cascades and erasing epigenetic marks

The fetal and early postnatal periods are the most vulnerable windows for neurogenesis and synaptic network formation. High levels of GCs and abnormal accumulation of the metabolite 2-HG, triggered by maternal prenatal stress, leave profound “epigenetic marks” on both mitochondria and the nucleus. This suppresses mitophagy, induces microglial overactivation, and causes aberrant synaptic pruning, ultimately increasing the risk of ASD.

Currently, effective early interventions for ASD mostly focus on correcting HPA axis overactivation. For example, sodium butyrate can significantly increase histone acetylation at the corticotropin-releasing hormone receptor 2 (CRHR2) promoter region, restoring the HPA axis negative feedback loop via epigenetic mechanisms (Wang X. et al., 2023). Furthermore, moderate pharmacological inhibition of the GR signaling pathway during pregnancy can restore normal astrocyte differentiation and alleviate ASD-like behavioral deficits in offspring (Yuan et al., 2026).

However, past studies on the direct role of 2-HG in neurodevelopment are limited. Recent evidence indicates that abnormal accumulation of 2-HG can epigenetically activate MYC (a core member of the bHLH transcription factor family), thereby blocking neuronal differentiation (Gu et al., 2026). Furthermore, in neuropathological models like amyotrophic lateralizing sclerosis (ALS), MYC-driven glial abnormalities severely disrupt neuron-glia communication (Fioretti et al., 2026). Therefore, targeting the abnormal production of 2-HG or blocking its downstream epigenetic activation of MYC holds promise as a novel therapeutic target. This approach could erase stress-induced “initial molecular marks” at the source, rescue synaptic development, and help treat early ASD.

### 6.2 Adolescence and depression: restoring mitochondrial dynamics and quenching retrograde ROS signals

Adolescence is a critical period for prefrontal cortex maturation, fine synaptic pruning, and the consolidation of emotion regulation networks. Chronic stress during this time specifically upregulates DRP1 expression, leading to excessive mitochondrial fission and fragmentation in neurons, accompanied by a massive accumulation of ROS. Acting as retrograde signals, these ROS not only induce nuclear epigenetic changes but also exacerbate excessive synaptic pruning by astrocytes, ultimately consolidating pathological depressive neural circuits.

Therapeutically, targeting the dynamic balance of DRP1 is a primary strategy for treating adolescent depression (Deng et al., 2022). Notably, abnormal ROS activation in adolescent mice is a core driver of impaired synaptic plasticity and neurobehavioral abnormalities (Zhang C. et al., 2025). Future antidepressant therapies should develop treatments targeting mitochondrial ROS. By cutting off this key “retrograde danger signal,” such treatments may effectively prevent aberrant synaptic engulfment by adolescent astrocytes (such as that mediated by MEGF10) and protect vulnerable affective and cognitive circuits.

### 6.3 Aging and PD/AD: unblocking lysosomal autophagic flux

Under conditions of aging and chronic stress accumulation, the “mitophagy-lysosome regulatory axis” in the CNS faces complete collapse. On one hand, lysosomal lipid overload prevents the degradation of autophagosomes. On the other hand, un-cleared damaged mitochondria release large amounts of mtDNA into the cytoplasm, activating the cGAS/STING pathway in microglia. This triggers a state of inflammaging, a phenomenon highly correlated with the accelerated clinical progression of neurodegenerative diseases like AD and PD.

Therapeutically, the core issue in the aging neurodegenerative brain lies in the stagnation of downstream lysosomal degradation. Using metabolic regulators such as Urolithin A can restore lysosomal acidification and degradation functions via caspase-related pathways. This process is crucial for clearing the abnormal accumulation of Aβ and Tau proteins and for alleviating AD symptoms (Hou et al., 2024).

Meanwhile, the link between mtDNA mutations and aging opens new avenues for delaying aging and treating related diseases (Xu X. et al., 2025). Studies confirm that enhancing mitophagy can significantly reduce the activation of the cGAS/STING pathway driven by intracellular free mtDNA (Jiménez-Loygorri et al., 2024). Therefore, in addition to repairing the autophagy-lysosome network, developing small-molecule drugs to specifically block mtDNA-cGAS binding, or inhibiting the overactivation of STING and its downstream pathways, could fundamentally reverse the microglial pro-inflammatory phenotype. This presents a highly promising new approach of “dual immune-metabolic intervention” for delaying aging and treating neurodegenerative diseases in the elderly.

## 7 Conclusion and perspective

This review focuses on how stress impacts the CNS through mitophagy across the lifespan. We highlight that psychological stress disrupts mitochondrial quality control through HPA-axis activation and glucocorticoid signaling, leading to persistent alterations in mitophagy across the lifespan. Meanwhile, signaling molecules released by damaged mitochondria—including 2-HG, ROS, and mtDNA—exert retrograde regulation over nuclear epigenetic landscapes.

We summarize the dynamic evolution of mitophagy in three key life stages: Fetal period--Maternal stress elevates GC levels, suppresses mitophagy, impairs neurodevelopment, and increases vulnerability to ASD and other neurodevelopmental disorders. Adolescence--Stress disturbs mitochondrial dynamics and mitophagy, disrupts synaptic remodeling, and raises the risk of affective disorders. Aging-The mitophagy–lysosome axis collapses, cumulative stress effects escalate, and susceptibility to AD, PD, and other neurodegenerative diseases rises sharply.

We further establish an integrated cell–age–stress three-dimensional regulatory framework involving neurons, astrocytes, and microglia under distinct age and stress conditions. Finally, we propose the lifespan intervention hypothesis, which emphasizes precision fine-tuning strategies during critical developmental windows, with strong potential for clinical translation.

Notably, this review delineates both the forward stress injury (mitophagy failure and damaged mitochondrial accumulation) and mitochondria-to-nucleus retrograde signaling. We emphasize the mechanisms by which unresolved mtDNA, 2-HG, and ROS translocate to the nucleus to drive epigenetic remodeling, clarifying the bidirectional regulatory axis underlying stress-mitochondria interactions.

However, several critical questions remain unresolved. The precise pathways by which damaged mitochondria transmit signals to the nucleus and direct epigenetic reprogramming are not fully defined. Identifying cell-specific and lifespan-specific sensors, effectors, and downstream epigenetic modifications remains a major challenge. More provocatively, whether this mechanism forms the basis of transgenerational inheritance remains a highly speculative hypothesis that warrants future multi-generational tracing studies.

Emerging evidence also indicates heightened vulnerability in regions such as the fetal temporal lobe and adolescent prefrontal cortex. A key unanswered question—and a central hypothesis for future research—is whether stress primarily impairs mitophagy in neurons within these critical circuits to directly compromise synaptic integrity, or whether initial damage targets specialized glia, whose subsequent dysfunction and inflammatory output drive regional network failure.

In summary, this review of the age-stratified stress–mitophagy–CNS axis establishes a key mechanistic framework centered on mitochondria–nucleus bidirectional regulation and introduces a novel three-dimensional regulatory model. Together, these insights provide promising directions for mechanistic research and future clinical translation.
